# Palmitoylethanolamide counteracts high-fat diet-induced gut dysfunction by reprogramming microbiota composition and affecting tryptophan metabolism

**DOI:** 10.3389/fnut.2023.1143004

**Published:** 2023-08-01

**Authors:** Claudio Pirozzi, Lorena Coretti, Nicola Opallo, Maria Bove, Chiara Annunziata, Federica Comella, Luigia Turco, Adriano Lama, Luigia Trabace, Rosaria Meli, Francesca Lembo, Giuseppina Mattace Raso

**Affiliations:** ^1^Department of Pharmacy, School of Medicine, University of Naples Federico II, Naples, Italy; ^2^Task Force on Microbiome Studies, University of Naples Federico II, Naples, Italy; ^3^Department of Clinical and Experimental Medicine, University of Foggia, Foggia, Italy; ^4^Department of Precision Medicine, University of Campania Luigi Vanvitelli, Naples, Italy

**Keywords:** obesity, gut-brain axis, N-acylethanolamines, serotonin, gut microbiota

## Abstract

Obesity is associated with gastrointestinal (GI) tract and central nervous system (CNS) disorders. High-fat diet (HFD) feeding-induced obesity in mice induces dysbiosis, causing a shift toward bacteria-derived metabolites with detrimental effects on metabolism and inflammation: events often contributing to the onset and progression of both GI and CNS disorders. Palmitoylethanolamide (PEA) is an endogenous lipid mediator with beneficial effects in mouse models of GI and CNS disorders. However, the mechanisms underlining its enteroprotective and neuroprotective effects still need to be fully understood. Here, we aimed to study the effects of PEA on intestinal inflammation and microbiota alterations resulting from lipid overnutrition. Ultramicronized PEA (30 mg/kg/die *per os*) was administered to HFD-fed mice for 7 weeks starting at the 12th week of HFD regimen. At the termination of the study, the effects of PEA on inflammatory factors and cells, gut microbial features and tryptophan (TRP)-kynurenine metabolism were evaluated. PEA regulates the crosstalk between the host immune system and gut microbiota via rebalancing colonic TRP metabolites. PEA treatment reduced intestinal immune cell recruitment, inflammatory response triggered by HFD feeding, and corticotropin-releasing hormone levels. In particular, PEA modulated HFD-altered TRP metabolism in the colon, rebalancing serotonin (5-HT) turnover and reducing kynurenine levels. These effects were associated with a reshaping of gut microbiota composition through increased butyrate-promoting/producing bacteria, such as Bifidobacterium, Oscillospiraceae and *Turicibacter sanguinis*, with the latter also described as 5-HT sensor. These data indicate that the rebuilding of gut microbiota following PEA supplementation promotes host 5-HT biosynthesis, which is crucial in regulating intestinal function.

## 1. Introduction

Intestinal homeostasis is preserved by multiple and complex interactions between gut microbiota and host immune system (1). This mutual relationship regulates many physiological functions strictly associated with metabolic and nutritional balance and immune system stimulation. Diets high in fat or sugar, antibiotic administration, and stress are known to induce, at different extents, dysbiosis, loss of gut integrity, and consequently inflammation, and an overall impact on host health. These events contribute to the onset of several diseases, not limited to the gastrointestinal (GI) tract, but involving extra-intestinal peripheral tissues, including the central nervous system (CNS) (2). Over the past two decades, many preclinical studies investigated the role of the gut-brain axis in obesity-induced GI inflammation and behavioral alterations (3, 4). Gut microbiota pivotally plays a role in gut-brain communication through several interrelated mechanisms, including the activation of afferent sensory neurons of the vagus nerve, neuro-immune and neuroendocrine pathways, microbial metabolites, and neurotransmitter release (5). One focal point in this regard is the microbial regulation of circulating tryptophan (TRP) levels, with a potential dual action in regulating serotonin (5-HT) synthesis and kynurenine (KYN) pathway metabolism (6), ultimately affecting both metabolic and neuropsychiatric disorders (7).

Palmitoylethanolamide (PEA), an endogenous lipid mediator belonging to the family of N-acylethanolamines (NAEs), has shown beneficial effects in colonic inflammatory conditions (8–10), and CNS diseases (11, 12). Therefore, the duality of enteroprotective and neuroprotective effects of PEA indicated novel lines of investigation on its potential effect on neurodegenerative and neurodevelopmental disorders through gut-brain axis involvement (13–15). Among CNS disorders, mood and cognitive alterations are often comorbidity during obesity (16). The mechanisms underlying this association involve inflammation, neurotransmitter unbalance, and overactivation of the hypothalamus-pituitary–adrenal (HPA) axis, with the microbiota functioning as a bridge between the brain and intestinal bidirectional communication (17).

We have recently demonstrated that PEA not only counteracts metabolic inflexibility and adipose tissue dysfunction in HFD-fed mice (18, 19) but also limits the depressive- and anxiety-like behaviors and the cognitive decline associated with high-fat feeding. The effects are associated with increased neurogenesis and synaptic strength and reduced neuroinflammation and blood–brain barrier disruption (20, 21). PEA also markedly modulates monoamine levels by decreasing dopamine (DA) levels and increasing DA turnover in the amygdala; by increasing 5-HT levels in the prefrontal cortex (PFC); and by reducing DA and 5-HT turnover in the nucleus accumbens and PFC, respectively (21). These findings, reinforced by the notion that PEA modulates gut microbiota composition and mood disorders (13, 22), prompted us to study the effect of chronic administration of PEA on gut dysfunction, microbiota composition and TRP metabolism induced by fat overnutrition.

## 2. Materials and methods

### 2.1. Animals and treatments

Six weeks-old C57Bl/6J male mice (Charles River, Wilmington, MA, USA) were housed in stainless steel cages in a room at 22 ± 1°C with a 12:12 h lights-dark cycle (from 7 a.m. to 7 p.m.). Mice were randomized and sorted into three groups (5/6 mice for each group) as follows: a control group (STD) receiving a chow diet (Mucedola srl, Milan, Italy) and vehicle; high-fat diet (HFD) (Research Diets Inc., NJ, USA) group receiving an HFD having 45% of energy derived from fat and 7% of sucrose vehicle. The exact composition of STD and HFD is summarized in Supplementary Table 1. HFD group treated with ultramicronized PEA (HFD + PEA, 30 mg/kg/die *per os*). The treatments began 12 weeks after HFD consumption and lasted 7 weeks concurrently with HFD. Ultramicronized PEA, provided by Epitech Group Labs (Padua, Italy) was dissolved in carboxymethylcellulose (1.5%) for oral administration. At the end of the study, mice were sacrificed by inhaled enflurane anesthesia followed by cervical dislocation and feces and colons were collected and stored at −80°C. All procedures involving animals complied with the Institutional Guidelines and according to the Italian D.L. no.116 of January 27, 1992 of Ministry of Health under the protocol no. 982/2017-PR, and associated guidelines in the European Communities Council Directive of November 24, 1986 (86/609/ECC).

### 2.2. Western blot analysis

Colon was homogenized in lysis buffer (20 mM Tris–HCl, pH 7,5, 10 mM NaF, 150 mM NaCl, 1% Nonidet P-40, 1 mM phenylmethylsulphonyl fluoride, 1 mM Na_3_VO_4_, leupeptin 10 μg/mL, and trypsin inhibitor 10 μg/mL). Total protein lysates were obtained as supernatant by centrifugation at 14,000 × g for 15 min at 4°C. Protein concentrations were estimated by the Bio-Rad protein assay using free bovine serum albumin (BSA) as standard. An equal amount of protein (40 μg) was subjected to SDS-PAGE and electro-transferred onto a nitrocellulose membrane using a Bio-Rad Transblot (Bio-Rad, Milan, Italy). Membranes were blocked at room temperature in milk buffer (1X PBS, 5% w/v non-fat dry milk) and probed with rabbit polyclonal antibody against anti-Toll like receptor 4 (TLR4) (1:1000; Invitrogen, Whaltam MA, USA; 48-2300; AB_2533842), rabbit polyclonal antibody anti-Cyclooxygenase (COX)-2 (1:500; Elabscience, Houston, TX; E-AB-31012; AB_2715578), mouse polyclonal antibody anti-indoleamine 2,3-dioxygenase (IDO) (1:500; Santa Cruz Biotechnology, Dallas, TX, USA; sc-137012; AB_2123436), mouse polyclonal antibody anti-inducible oxide nitric synthase (iNOS) (1:1000; BD Biosciences, Franklin Lakes, NJ, USA; 610432; AB_397808). Western Blot for anti-β-Actin (1:8000; Sigma-Aldrich St. Louis, MO, USA; A5441; AB_476744) was performed to ensure equal sample loading and data were expressed as relative normalized expression. The filter detection was performed by ChemiDoc Imaging System (Bio-Rad Laboratories, Hercules, CA, USA).

### 2.3. RNA extraction and semi-quantitative real-time PCR (RT-PCR)

Total RNA isolated from the colon was extracted using TRIzol Reagent (Bio-Rad Laboratories, Hercules, CA, USA; 7326890) following the extraction kit’s protocol for RNA (NucleoSpin^®^, Macherey-Nagel GmbH & Co, Düren, Germany; FC140955N). cDNA was obtained using High-Capacity cDNA Reverse Transcription Kit (Applied Biosystems, Foster City, CA, USA; 4374966) from 8 μg total RNA. RT-PCRs were performed with a Bio-Rad CFX96 Connect Real-time PCR System instrument and software (Bio-Rad Laboratories). The RT-PCR conditions were 15 min at 95°C followed by 40 cycles of two-step PCR denaturation at 94°C for 15 s, annealing extension at 55°C for 30 s and extension at 72°C for 30 s, as previously described (23). Each sample contained 500 ng cDNA in 2X QuantiTect SYBR Green PCR Master Mix (204145) and primers pairs to amplify chymase 1 (*Cma1*; QT0019946), tryptase β2 (*Tpsb2*; QT00252637), IL-1β (*Il1b*; QT01048355), TNF-α (*Tnf*; QT00104006), integrin αX subunit (*Itgax*; QT00113715), EGF-like module-containing mucin-like hormone receptor-like 1 (*Emr1*; QT00099617), corticotropin-releasing hormone (*Crh*; QT00293489), free fatty acid receptor 2 (*Ffar2*; QT00128226) and solute carrier family 16 member 1 (*Slc16a1*; QT00115423) (Qiagen, Hilden, Germany), in a final volume of 50 μL. The relative amount of each studied mRNA was normalized to *Gapdh* (QT01658692) (Qiagen, Hilden, Germany) as a housekeeping gene, and data were analyzed according to the 2^−∆∆CT^ method.

### 2.4. High-performance liquid chromatography (HPLC) quantifications

Determination of 5-HT, 5-hydroxyindolacetic acid (5-HIAA) and KYN levels in colon were performed by using HPLC coupled with an electrochemical detector (Ultimate ECD, Dionex Scientific, Milan, Italy), as previously described (24). Briefly, colon samples were homogenized and separated by a LC18 reverse phase column (Kinetex, 150 mm × 4.2 mm, ODS 5 μm; Phenomenex, Castel Maggiore-Bologna, Italy). Analytes were detected through a thin-layer amperometric cell (Dionex, ThermoScientifics, Milan, Italy) with a 5 mm diameter glassy carbon electrode at a working potential of 0,400 V (vs. Pd) for 5-HT and 5-HIAA, and 0.750 V (vs. Pd) for KYN, with a flow rate of 0.7 mL/min by using an isocratic pump (Shimadzu LC-10 AD, Kyoto, Japan). A solution of 75 mM NaH2PO4, 1.7 mM octane sulfonic acid, 0.3 mM EDTA, acetonitrile 10%, in distilled water, buffered at pH 3.0, was used as mobile phase. All reagents were purchased from Sigma Aldrich, Milan Italy. Data analysis was accomplished by Chromeleon software (version 6.80, Thermo Scientific Dionex, San Donato Milanese, Italy).

### 2.5. Microbiota sequencing and data analysis

Fecal microbiota of STD, HFD, and HFD + PEA groups was analyzed by collecting samples from a subset of 4/5 mice/group and immediately stored at −80°C until processed. Bacterial genomic DNA was extracted from frozen fecal samples using the QIAamp DNA Stool Mini Kit (Qiagen) according to the manufacturer’s instructions. Extracted DNA concentration was measured fluorometrically using Qubit dsDNA BR assay kit (Invitrogen) and quality was checked by spectrophotometric measurements with NanoDrop (ThermoFisher Scientific Inc). The V3-V4 region of the 16S rRNA gene was amplified and prepared for sequencing according to the protocol 16S Metagenomic Sequencing Library Preparation for Illumina Miseq System as previously described (25). Sequencing run was performed on the Illumina MiSeq system using v3 reagents for 2 × 281 cycles (Illumina, Inc.). Metataxonomic analysis was conducted using the Quantitative Insights Into Microbial Ecology (QIIME2, version 2021.8) (26). V3-V4 16S rDNA FASTQ paired-end reads were quality filtered, dereplicated, denoised, merged, and assessed for chimeras using DADA2 pipeline (27). Amplicon sequence variants (ASVs) were obtained and filtered out based on a prevalence of at least two samples. Taxonomic assignment was performed utilizing SILVA v138 database, with a classifier trained on the amplified regions (28). Moreover, to achieve a better species taxonomic level resolution, each ASV sequence has also been aligned to a taxonomy classifiers from NCBI Genbank (29, 30). A rarefaction procedure was performed to assess sampling depth coverage and species heterogeneity in each sample. Sample size biases in subsequent analyses were avoided by applying a sequence rarefaction procedure using a depth of 9,151 reads/sample. Alpha diversity within each group was assessed by calculating the following parameters: Observed features, Chao1 (to assess species richness), Shannon’s and Simpson (as a measure of species distribution) (31). The statistical significance of alpha diversity was assessed by the Kruskal-Wallis test. Diversity among sample communities was detected by performing beta diversity analysis calculating weighted and unweighted UniFrac distance matrices (32, 33). The statistical significance of beta diversities was assessed on phylogenetic distance matrixes using the ANOSIM method with 999 permutations. Firmicutes/Bacteroidota ratio was also calculated and statistical differences among groups were evaluated through one-way ANOVA followed by Tukey multiple comparison post-hoc tests. The linear discriminant analysis effect size (LEfSe) method was used to identify key species of each group (LDA score > 2; *p* < 0.05).^1^

### 2.6. Statistical analysis

All data shown are presented as mean value ± SEM. Statistical analysis was performed by one-way ANOVA, followed by Bonferroni *post hoc* for multiple comparisons. Differences among groups were considered significant at values of *p* < 0.05. Analyses were performed using GraphPad Prism 9 (GraphPad Software, San Diego, CA, USA).

## 3. Results

### 3.1. PEA reduces immune cells infiltration and stress marker in colon of HFD-fed mice

**Figure 1:**
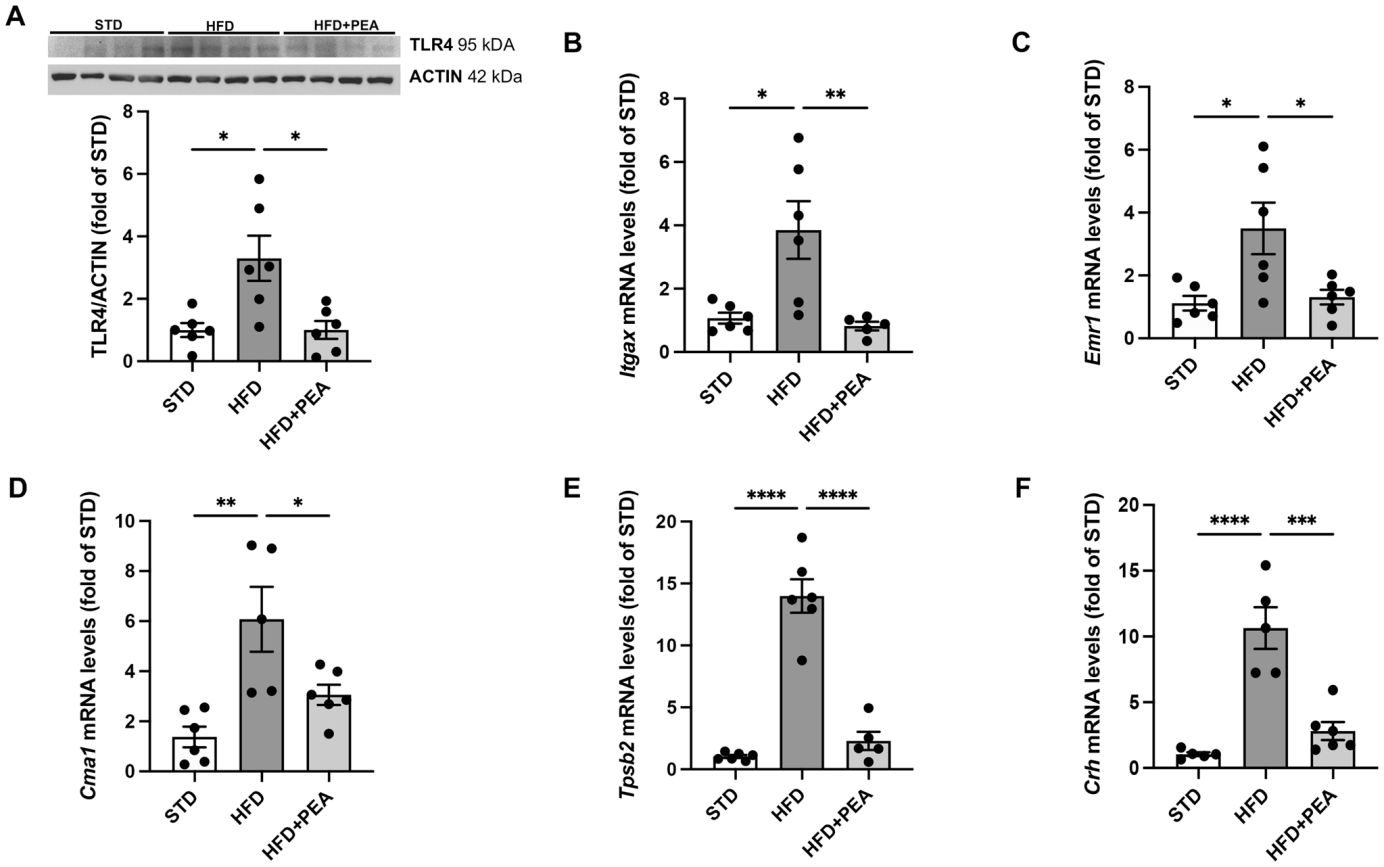
PEA counteracts immune cell infiltration caused by HFD overfeeding. **(A)** Protein levels of TLR4 were evaluated by Western Blot analysis. **(B–F)** PEA normalized mRNA levels of *Itgax*, *Emr1*, *Cma1*, *Tpsb2* and *Crh* in the colon of HFD group (*n* = 5–6 each group). A representative Western blot is shown for TLR4. Data are presented as mean ± SEM reaching the significance at *P* < 0.05 (*P < 0.05, **P < 0.01, ***P < 0.001, and ****P < 0.0001).

HFD-fed mice showed a significant increase in protein expression of TLR4, a major player in the immune response, compared with STD and HFD + PEA groups (Figure 1A). Furthermore, PEA reduced the transcriptional levels of *Itgax* (Figure 1B), an integrin mainly expressed by macrophages, monocytes, and NK cells. The levels of *Emr1* (Figure 1C), a murine marker of macrophages markedly upregulated by HFD, were also reduced in the HFD + PEA group. PEA treatment also counteracted HFD-induced mast cell activation, reducing gene expression of murine-specific chymase-1 and tryptase β2 (Figures 1D,E). Moreover, we evaluated *Crh* transcription as a marker of psychogenic stress, showing PEA capability in reducing its expression in the colon (Figure 1F).

### 3.2. PEA reduces intestinal inflammation in HFD-fed mice

**Figure 2:**
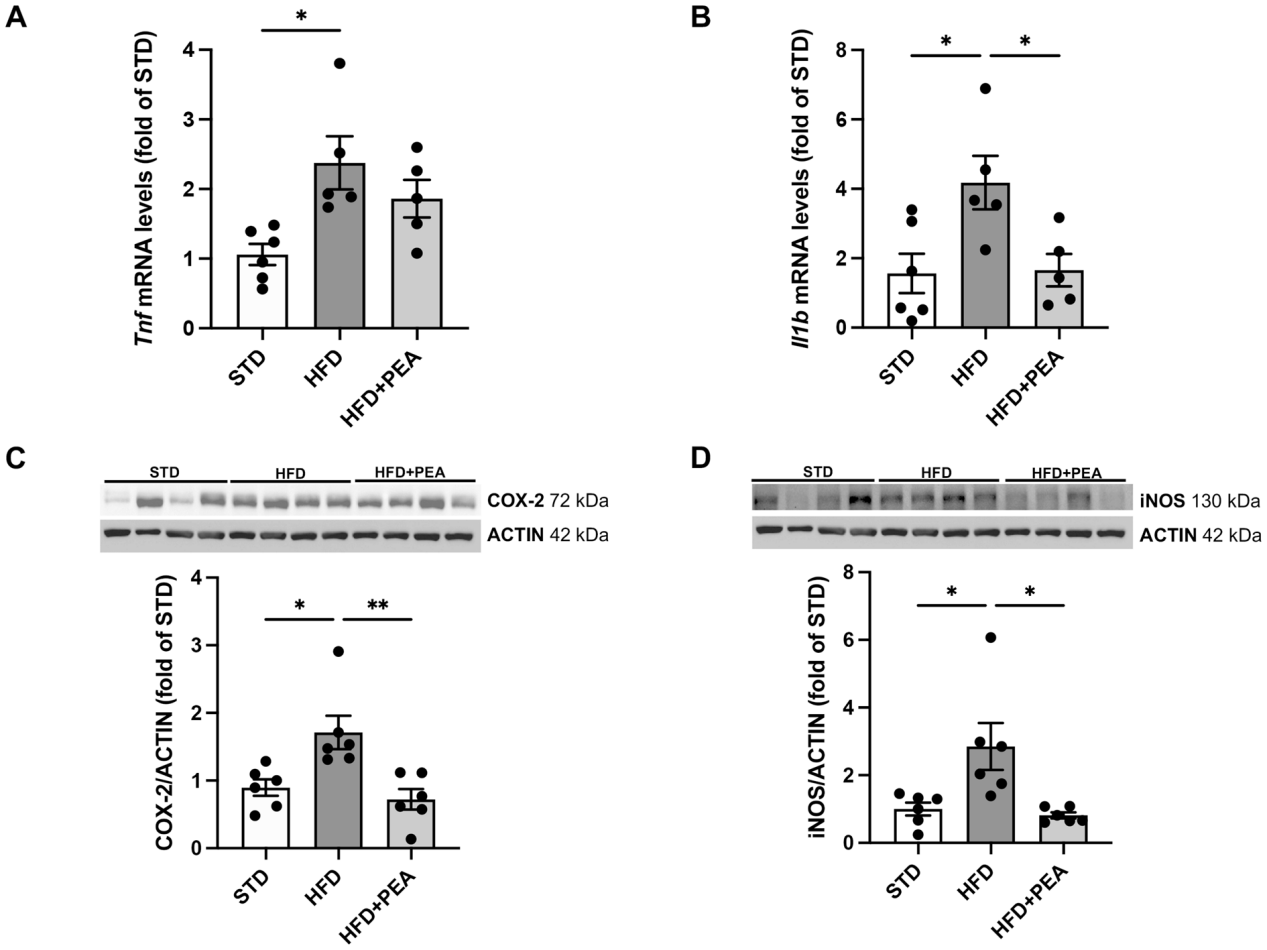
PEA lessens gut inflammation enhanced by HFD. **(A,B)** Transcriptional levels of inflammatory cytokines, *Tnf* and *Il1b*, were assessed in colonic tissue. **(C,D)** PEA modulated protein levels of COX-2 and iNOS (*n* = 5–6 each group). Representative Western blots are shown for COX-2 and iNOS. Data are showed as mean ± SEM reaching the significance at *P* < 0.05 (*P < 0.05, **P < 0.01).

As shown in Figure 2, HFD feeding caused an increase in the levels of proinflammatory cytokines, such as TNF-α and IL-1β (Figures 2A,B), and the protein expression of iNOS and COX-2 (Figures 2C,D) in colonic tissues. The treatment with PEA dampened gut inflammation, significantly reduced the main proinflammatory factors, and partially lowered TNF-α mRNAs.

### 3.3. PEA modulates gut serotonin levels and IDO/kynurenine pathway altered by HFD

**Figure 3:**
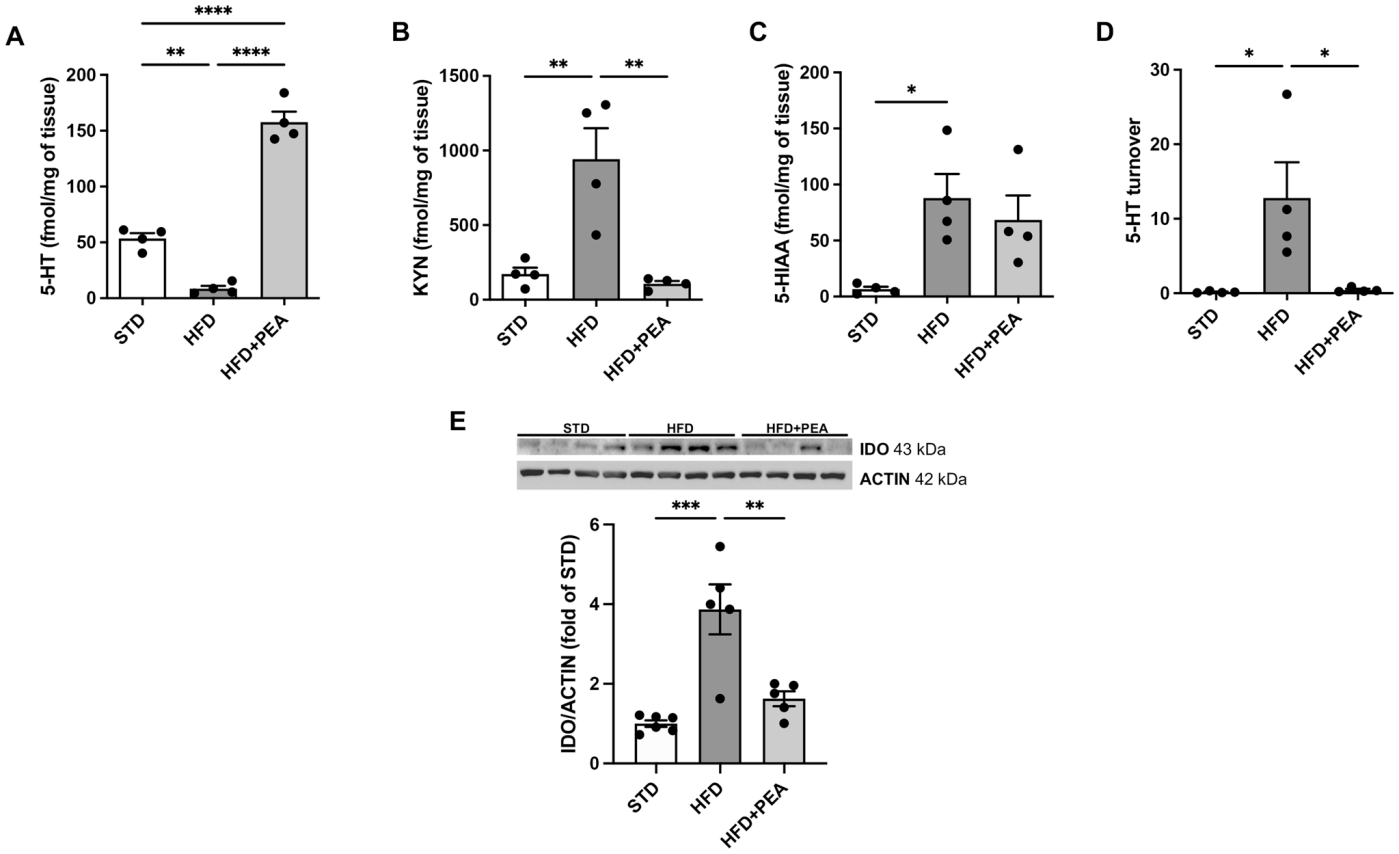
PEA increases serotonin levels and modulates IDO/kynurenine pathway in the gut. **(A–D)** PEA increased 5-HT levels in the colon, also modulating KYN, 5-HIAA, and 5-HT turnover (*n* = 4 each group). Data are obtained by HPLC. **(E)** Protein expression of IDO, increased by HFD overfeeding, was normalized by PEA treatment (*n* = 5–6 each group). A representative Western blot is shown for IDO. Data were showed as mean ± SEM reaching the significance at *P* < 0.05 (*P < 0.05, **P < 0.01, ***P < 0.001, and ****P < 0.0001).

HFD feeding caused a significant decrease in 5-HT in the colon of obese mice. The high levels of the metabolite 5-HIAA in the HFD group compared to STD suggest an increased metabolism of this neurotransmitter in the gut (Figures 3A,C). Moreover, an increased amount of KYN was measured in colon tissues from obese mice and the induced protein expression of IDO (Figures 3B,E). PEA treatment induced an increase in the levels of 5-HT, paralleled by a reduction of its turnover (Figure 3D) and KYN levels, possibly through the downregulation of IDO protein expression.

### 3.4. PEA reshapes gut microbiota composition in obese mice

High-throughput sequencing targeting the V3-V4 regions of the 16S rRNA gene was used to examine the effects of PEA treatment on gut microbiota alterations induced by HFD. Following sequence denoising, trimming and chimera picking, 1,166 different features (ASVs with ≥2 counts) were inferred from a total of 301,699 reads. Data were rarefied to the minimum library size of 9,151 reads/sample, a sequencing depth considered adequate as all curves reached ASV detection saturation (data not shown).

**Figure 4:**
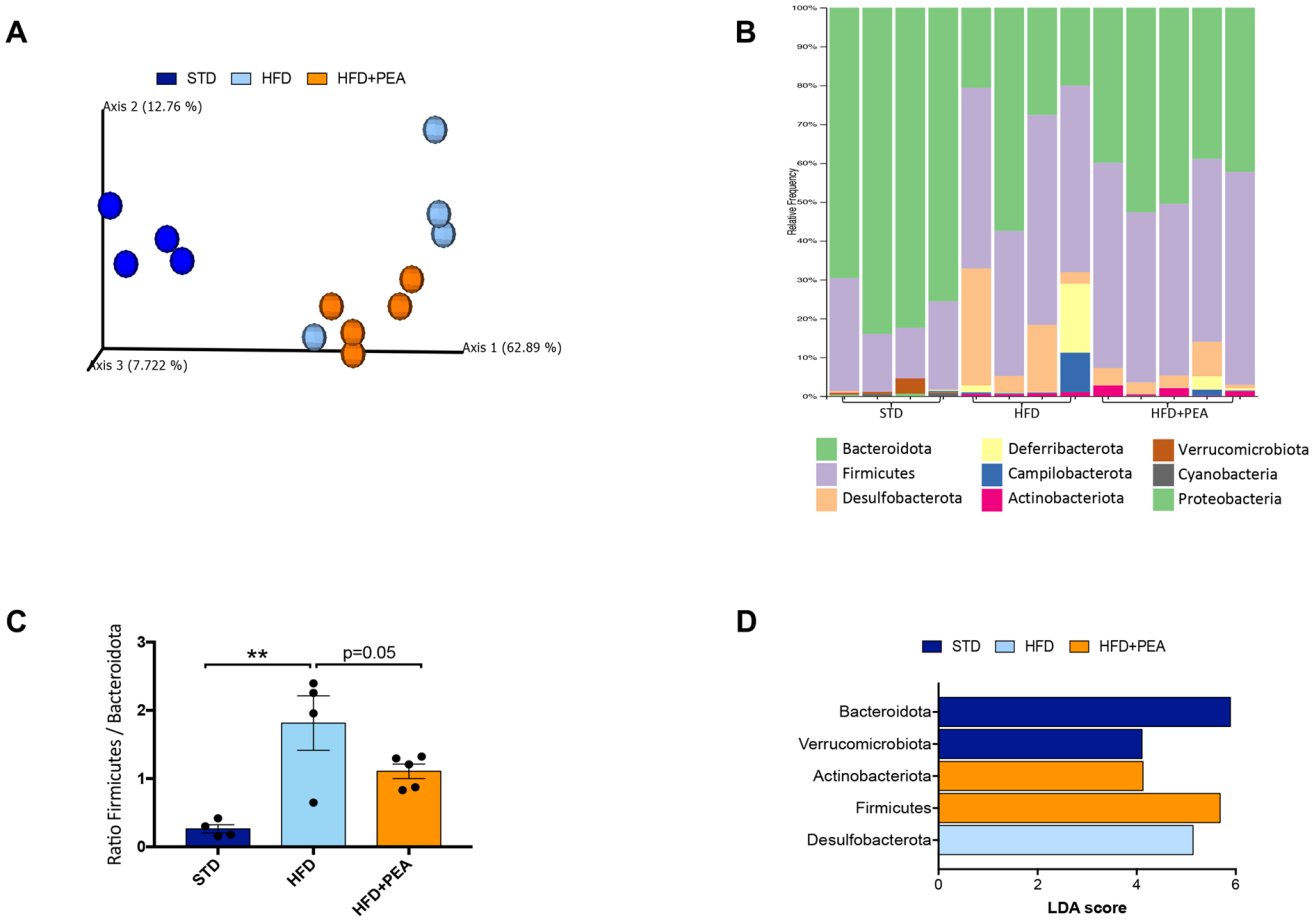
HFD mice microbial communities upon PEA supplementation. **(A)** Principal coordinate analysis (PCoA) plot based on weighted UniFrac distance matrix (9,151 reads/sample). **(B)** Stacked bar chart showing the sample relative abundance of all bacterial ASVs taxonomically classified at phylum level. **(C)** Firmicutes to Bacteroidota ratio in each sample group (mean ± SEM, **P < 0.01, one-way ANOVA followed by Tukey’s *post hoc* test for multiple comparisons). **(D)** Gut microbiota differences at phylum taxonomic level based on linear discriminant analysis (LDA) combined with effect size (LEfSe) algorithm (*P* > 0.05 for both Kruskal–Wallis and pairwise Wilcoxon tests and a cutoff value of LDA score above 2.0).

Principal coordinates analysis (PCoA) based on weighted UniFrac distances showed that HFD changed the composition of the gut microbiota (ANOSIM HFD vs. STD: *R* = 0.92 and *p* = 0.02) and revealed that the fecal bacterial community of PEA-treated HFD mice diverged from that of untreated HFD mice (ANOSIM R = 0.29 and *p* = 0.046). However, a strong difference rather than STD was still observable (ANOSIM *R* = 1 and *p* = 0.01) (Figure 4A).

**Figure 5:**
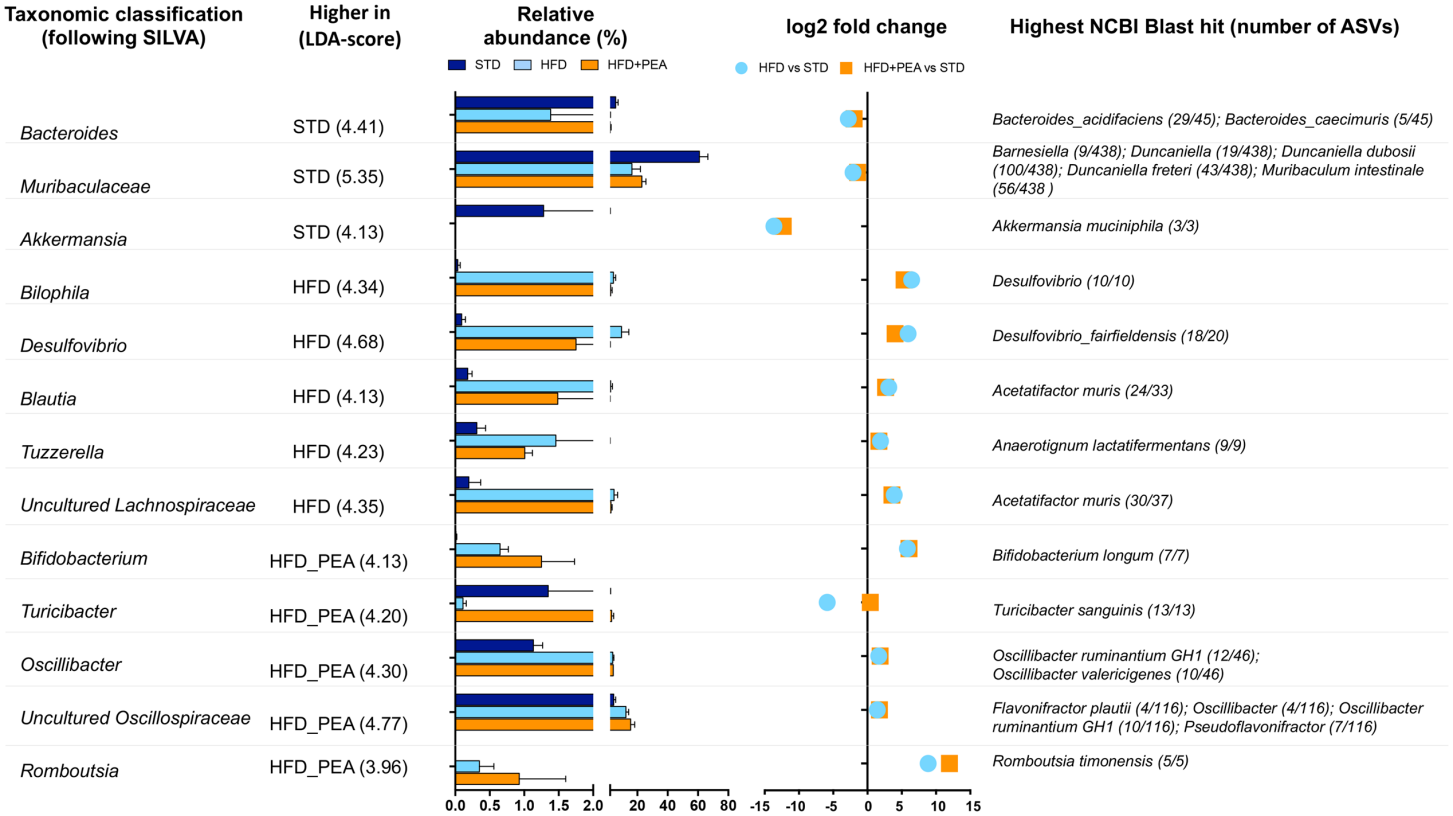
Significantly changes genera in HFD and HFD + PEA mice. Significantly changed genera were identified using LEfSe algorithm [alpha values of 0.05 for both Kruskal–Wallis and pairwise Wilcoxon tests and a cutoff value of LDA score (log10) above 2.0]. For each key genus, LDA score, relative abundance (mean and STD err), log2 transformed fold change in HFD and HFD + PEA compared to STD levels, and highest NCBI Blast hits are reported.

The gut microbiota composition was then studied at phylum and genus taxonomic levels (Figures 4B–D, 5). For an accurate taxonomic classification and species-level profiling, the representative sequence of each ASV belonging to key genera was re-annotated according to the NCBI taxonomy (Figure 5). At the phylum level, Firmicutes and Bacteroidota phyla dominated the microbial communities of all groups (Figure 4B); however, Firmicutes/Bacteroidota ratio significantly increased in HFD mice principally due to 31.49% of Bacteroidota and 46.37% of Firmicutes compared with 77.72 and 20.07% in STD mice for Bacteroidota and Firmicutes, respectively. Notably, PEA supplementation restored Firmicutes/Bacteroidota ratio to STD levels (Figure 4C). LEfSe algorithm, applied to identify the key feature marking the fecal microbiota of each group, indicated phylum Desulfobacterota significantly enriched in HFD mice and phyla Actinobacteria and Firmicutes in PEA-treated mice. Conversely, Bacteroidota and Verrucomicrobia were reduced in HFD and HFD + PEA groups compared to STD mice (Figure 4D). Furthermore, the statistical analysis highlighted, at the genus level, a selection for members of the gut bacterial communities upon HFD and PEA treatment. Different bacterial genera, namely *Bilophila*, *Desulfovibrio* (*D. fairfieldensis*), *Blautia* (closely related to *Acetatifactor muris*), *Tuzzerella* (*Anaerotignum lactatifermentans*) and an uncultured genus belonging to Lachnospiraceae family (closely related to *Acetatifactor muris*), were significantly increased in the HFD group compared with both STD and HFD + PEA groups; in contrast, *Bifidobacterium* (*B. longum*), *Turicibacter* (*T. sanguinis*), *Romboutsia* (*R. timonensis*), *Oscillibacter* (closely related to *Oscillibacter ruminantium* GH1 and *Oscillibacter valericigenes*), and several members of Oscillospiraceae family were significantly enriched in PEA-supplemented HFD mice (Figure 5). Figure 5 also reports the fold change of each key genus in HFD and PEA-treated mice compared to the STD group. Notably, in the HFD + PEA group, it was possible to detect an enhancement of bacteria already increased by HFD (e.g., *Bifidobacterium*, *Rombustia*, and *Oscillibacter*) but mainly a trend of restoration to standard levels of the majority of the bacteria genera affected by HFD (such as *Bacteroides*, *Muribaculaceae*, *Bilophila*).

### 3.5. PEA induces the receptor (GPR43) and transporter (MCT1) of butyrate in colon of HFD mice

**Figure 6:**
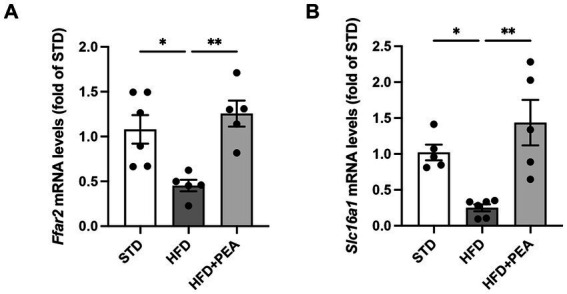
PEA induces the transcription of colonic butyrate receptor and transporter. mRNA levels of **(A)** *Ffar2* and **(B)** *Slc16a1* were reduced by HFD and restored by PEA treatment in the colon of obese mice (*n* = 5–6 each group). Data were showed as mean ± SEM reaching the significance at *P* < 0.05 (*P < 0.05 and **P < 0.01).

Since the possible modulation of butyrate-producers bacteria induced by PEA treatment, mRNA levels of butyrate receptor (*Ffar2*) and transporter (*Slc16a1*) were evaluated in the colon of mice (Figures 6A,B). After 19  weeks of HFD, obese mice showed a significant reduction of the transcription of both parameters that were increased by PEA treatment.

## 4. Discussion

**Figure 7:**
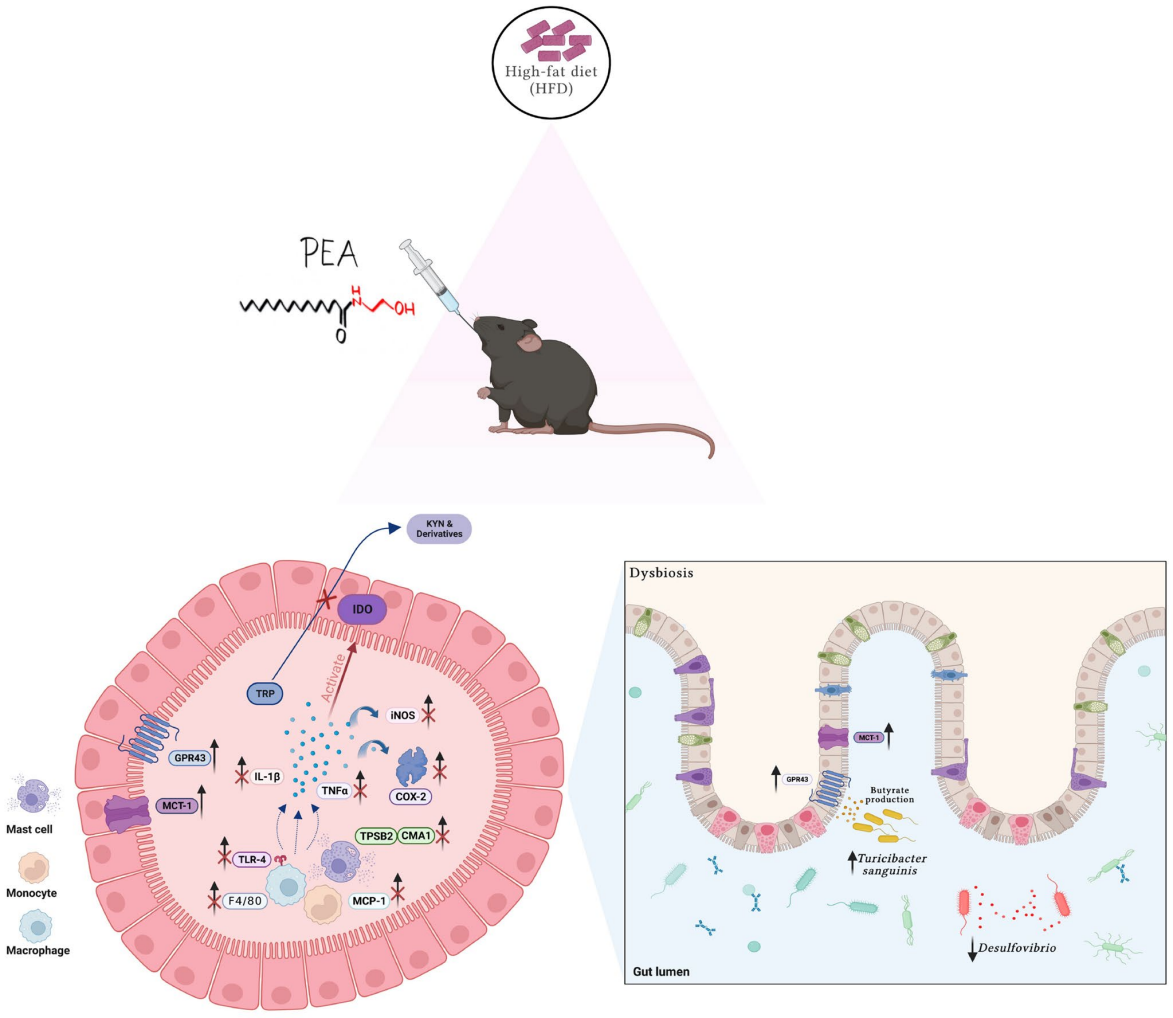
Summarizing figure of PEA activity in the colon of HFD mice. Multiple effects of PEA on gut inflammation, TRP metabolites and microbiota composition. Created with biorender.com.

In this study, we showed that PEA exerts protective effects in long-term HFD-induced intestinal damage in mice, modulating gut microbiota composition and restoring tryptophan-derived metabolites altered by HFD (Figure 7).

Despite the lack of evidence regarding the effect of PEA on obesity-related gut dysfunction, previous studies have demonstrated its beneficial effects at the GI level (34, 35). Several NAEs, including PEA and oleoylethanolamide, inhibit intestinal hypermotility and attenuate the inflammatory and the immune response through multiple converging mechanisms, e.g., the activation of the peroxisome proliferator-activated receptor (PPAR)-α (36, 37). Moreover, endogenous NAE levels changed in the GI tract in response to several noxious stimuli to regulate food intake, energy balance, and intestinal function (38).

The gut is the earliest and primary source of the inflammatory process triggered by fat overnutrition, based on direct exposure to dietary-derived components (39). The lack of gut barrier integrity, undermined by HFD, induces endotoxemia and contributes to the low-grade systemic inflammation. Thus, TLR4 activation by LPS and fatty acids represents a key link between the inflammatory process and the immune response in the gut during high-fat feeding (40). Indeed, TLR4 knockout mice have attenuated HFD-induced systemic or intestinal inflammation (41). Here, consistently with our previous finding showing that PEA reduces circulating LPS levels in HFD-fed mice (21), we report a decreased protein expression of colonic TLR4, associated with reduced immune cell markers (i.e., *Itgax*, *Emr1*, *Cma1*, and *Tpsb2*). These data indicate that the oral administration of ultramicronized PEA has immunomodulatory effects in obese mice, limiting immune cell recruitment and mast cell activation. Intestinal mast cells represent a crucial neuroimmune defense mechanism at the frontline between the host and the environment (42). Psychogenic stressors, including fat overnutrition, stimulate mast cell degranulation, leading to the release of histamine and tryptase in the gut of both humans and animals (43–45). In our study, PEA administration reduces not only colonic expression of chymase and tryptase, but also *Crh* transcription, indicating its capability to blunt stress-related gut function alterations. Notably, doxantrazole, a mast cell stabilizer, limited CRH-induced hypersensitivity of the colon of maternally separated rats (46), highlighting the detrimental role of mast cell activation at colonic level in stress-related conditions.

The reduced expression of immune cell markers by PEA treatment, particularly regarding macrophages and mast cells, is markedly consistent with the reduction of inflammatory factors in the gut, namely *Il1b* mRNA, and COX-2 and iNOS protein expression.

Proinflammatory factors and cytokines are known to affect tryptophan-kynurenine pathway and its final products. These metabolites sustain local inflammation, foster GI disorders, and may be involved in the pathogenesis of numerous central diseases. TRP metabolism stands in the bridge between the gut and CNS (47). Most of the TRP is oxidized into KYN by the rate-limiting enzyme indoleamine-2-3 dioxygenase (IDO), mainly expressed in the brain, GI tract, and liver, or by tryptophan 2,3-dioxygenase (TDO) explicitly expressed in the liver. KYN can be further metabolized through two divergent pathways associated with the synthesis of kynurenic acid or quinolinic one, whose ratio modulates diverse pathophysiological processes at both central and GI levels. Beyond the IFN-γ-dependent pathway, IDO activity is also synergistically stimulated by the crosstalk among TLR4, IL-1 receptor (IL-1R) and TNF-α receptor (TNFR), increasing the overall KYN levels, which can be transported into the brain and trigger detrimental changes (48). Notably, the enhanced activity of KYN pathway and simultaneous reduction in 5-HT levels have been associated not only with depressive-, anhedonic-, and anxiety-like behavior (49) but also with metabolic dysfunctions related to obesity and insulin resistance (50). Here, we show that PEA re-establishes 5-HT/KYN levels at the colonic level, restoring the altered 5-HT turnover and reducing KYN levels and IDO expression.

It is conceivable to hypothesize that KYN, whose gut levels are increased in HFD mice, reaches the systemic circulation, crosses the blood–brain barrier, and affects brain functions, contributing to behavioral patterns. As already demonstrated, HFD-fed mice show depressive- and anxiety-like phenotypes that are counteracted by administration of PEA (20, 21).

Obesity and associated metabolic disorders show altered gut microbiota composition, impacting on overall human health (51, 52). Thus, microbial reshaping has been proposed as a druggable target. Here, we show that the administration of ultramicronized PEA reprograms gut microbial community assortment. We propose that this event may be considered as a further mechanism in attenuating HFD-induced disorders. According to previous studies, HFD feeding disturbed microbiota homeostasis by increasing Firmicutes/Bacteroidota ratio, a feature associated with obesity and other related metabolic conditions (53). In this study, HFD feeding decreases explicitly the prevalence of the gut barrier-protecting species *Akkermansia muciniphyla* and increases the prevalence of Desulfobacterota, namely *Bilophila* and *Desulfovibrio* genera, opportunistic pathogens, and sources of LPS (54, 55). Desulfobacterota genera are positively related to metabolic disorders, such as type 2 diabetes (56, 57), and their hydrogen sulfide generation might induce intestinal barrier dysfunction and chronic inflammation (58). PEA reduced Firmicutes/Bacteroidota ratio, partially counteracting the HFD-induced increase of specific genera and raising the abundance of those profoundly decreased in the HFD group. PEA treatment also augments the levels of genera already increased by HFD, such as *Bifidobacterium* and Oscillospiraceae members that are potentially butyrate-producing/promoting bacteria (59–63). The further expansion of these bacteria in PEA-treated obese mice could imply beneficial effects on the overall intestinal environment. It may compensate for the loss of beneficial microbes, such as *Akkermansia*, resulting in a healthier gut in terms of mucus layer integrity and reduced inflammation.

Furthermore, PEA increased the relative abundance of *Turicibacter sanguinis,* a spore-forming microbe and short-chain fatty acids (SCFAs)-producer that decreases in obese rodents and alters the expression of gene pathways crucial for lipid and steroid metabolism (64–66). Recently, *T. sanguinis* has been proposed as a serotonin sensor, promoting host 5-HT biosynthesis (66). In this context, *T. sanguinis* could impact the amount of 5-HT synthesized and secreted by enterochromaffin cells throughout its butyrate-generation aptitude. The increased production of SCFAs has been associated with many beneficial effects, including amelioration of obesity and insulin resistance (67). A key role in gut homeostasis has been addressed to butyrate, which is endowed with profound protective effects related to the reversal of obesity and insulin resistance (68). Notably, colon tissue from PEA-treated mice revealed an increase in the expression of genes related to butyrate activity, e.g., MCT1, which mediates butyrate transport into the colonic mucosa, and GPR43 involved in the control of gut inflammation, indicating an increased sensitivity to local butyrate production.

Moreover, in our study, the increased relative abundance of *T. sanguinis* is associated with higher levels of serotonin content in PEA-treated obese mice. We hypothesize that the reshaping of gut microbiota structure and PEA supplementation might concomitantly promote host 5-HT biosynthesis and rebalance the TRP-KYN metabolism. In conclusion, our results revealed that oral administration of ultramicronized PEA restores colonic homeostasis associated with the reshaping of gut microbiota, the rebalance of TRP-KYN metabolism, and the reduction of the inflammatory response in obese mice, strongly supporting our hypothesis that PEA may have beneficial effects on CNS comorbidities via the gut-brain axis.

## Data availability statement

The sequences reported in this study are deposited in the ‘European Nucleotide Archive’ under the accession number PRJEB61012.

## Ethics statement

The animal study was reviewed and approved by Italian D.L. no. 116 of January 27, 1992 of Ministry of Health under the protocol n. 982/2017-PR, and associated guidelines in the European Communities Council Directive of November 24, 1986 (86/609/ECC).

## Author contributions

CP, CA, FC, NO, MB, LTu, and AL performed the experiments. CP, LC, and AL performed the research and data analysis. LTr and RM according to their expertise, analyzed data, and reviewed the manuscript. CP, AL, FL, and GM analyzed data and wrote the manuscript. CP, AL, and GM conceived the study design. All authors contributed to manuscript revision, read, and approved the submitted version.

## Funding

This work was partially supported by a grant assigned to RM from Epitech Group S.p.A. The funding organization had no influence on: (1) the study design, (2) the collection, analysis, and interpretation of data; (3) the writing of the manuscript; and (4) the decision to submit the manuscript for publication. This work was also partially supported by #NEXTGENERATIONEU (NGEU) and funded by the Ministry of University and Research (MUR), National Recovery and Resilience Plan (NRRP), project MNESYS (PE0000006) – A Multiscale integrated approach to the study of the nervous system in health and disease (DN. 1553 11.10.2022).

## Conflict of interest

AL declares that he has benefited from a fellowship supported by Epitech Group S.p.A.

The remaining authors declare that the research was conducted in the absence of any commercial or financial relationships that could be construed as a potential conflict of interest.

## Publisher’s note

All claims expressed in this article are solely those of the authors and do not necessarily represent those of their affiliated organizations, or those of the publisher, the editors and the reviewers. Any product that may be evaluated in this article, or claim that may be made by its manufacturer, is not guaranteed or endorsed by the publisher.

## Footnotes

^1^ https://huttenhower.sph.harvard.edu/galaxy
