# Supplementary material for: Palmitoylethanolamide counteracts high-fat diet-induced gut dysfunction by reprogramming microbiota composition and affecting tryptophan metabolism
Source: Front Nutr. 2023 Aug 1;10:1143004. doi: 10.3389/fnut.2023.1143004 (PMC10434518; doi:10.3389/fnut.2023.1143004)
Supplement: Supplementary file 1 [file Table_1.DOCX]

Palmitoylethanolamide counteracts high-fat diet-induced gut dysfunction by reprogramming microbiota composition and affecting tryptophan metabolism

**Claudio Pirozzi^1^, Lorena Coretti^1,2^, Nicola Opallo^1^, Maria Bove^3^, Chiara Annunziata^1^, Federica Comella^1^, Luigia Turco^1,4^, Adriano Lama^1,2,3*^, Luigia Trabace^3^, Rosaria Meli^1^, Francesca Lembo^1,2^, Giuseppina Mattace Raso^1,2^**

^1^Department of Pharmacy, School of Medicine, University of Naples Federico II, Naples, Italy

^2^Task Force on Microbiome Studies, University of Naples Federico II, Naples, Italy

^3^Department of Clinical and Experimental Medicine, University of Foggia, Foggia, Italy,

^4^Department of Precision Medicine, University of Campania Luigi Vanvitelli, Naples, Italy.

*** Correspondence:**Adriano Lama, PhD

University of Naples Federico II, Department of Pharmacy, Via Domenico Montesano 49, Naples, Italy.

adriano.lama@unina.it

|  | **STD** | **HFD** |
| --- | --- | --- |
| **Diet composition** | **%** | **%** |
| **Protein** | 29 | 21,2 |
| **Carbohydrate** | 60,4 | 24 |
| **Fat** | 10,6 | 54,8 |
| **Energy, kJ/g** | 15,88 | 21,9 |

# Supplementary Table

**Supplementary Table 1.** Composition formulas and fatty acid profile of STD (Mucedola s.r.l., Milan, Italy) and HFD diet (Research Diets Inc., New Brunswick, NJ, USA).
